# RS-5645 attenuates inflammatory cytokine storm induced by SARS-CoV-2 spike protein and LPS by modulating pulmonary microbiota

**DOI:** 10.7150/ijbs.63329

**Published:** 2021-07-25

**Authors:** Te Liu, Jianchao Wu, Changpeng Han, Zhangbin Gong, Giuseppe La Regina, Jiulin Chen, Fangfang Dou, Romano Silvestri, Chuan Chen, Zhihua Yu

**Affiliations:** 1 Shanghai Geriatric Institute of Chinese Medicine, Shanghai University of Traditional Chinese Medicine, Shanghai 200031, China.; 2 Yueyang Hospital of Integrated Traditional Chinese and Western Medicine, Shanghai University of Traditional Chinese Medicine, Shanghai, China.; 3 Department of Biochemistry, College of Basic Medicine, Shanghai University of Traditional Chinese Medicine, Shanghai 200031, China.; 4 Laboratory affiliated with the Institute Pasteur Italy-Cenci Bolognetti Foundation, Department of Drug Chemistry and Technologies, Sapienza University of Rome, Rome, Italy.

**Keywords:** SARS-CoV-2 (COVID-19) spike protein, 4-(thiophen-3-yl)-1-(p-tolyl)-1H-pyrrol-3-yl)(3,4,5-trimethoxyphenyl)methanone (RS-5645), inflammatory factor storm, pulmonary microbiota

## Abstract

An inflammatory cytokine storm is considered an important cause of death in severely and critically ill COVID-19 patients, however, the relationship between the SARS-CoV-2 spike (S) protein and the host's inflammatory cytokine storm is not clear. Here, the qPCR results indicated that S protein induced a significantly elevated expression of multiple inflammatory factor mRNAs in peripheral blood mononuclear cells (PBMCs), whereas RS-5645 ((4-(thiophen-3-yl)-1-(p-tolyl)-1H-pyrrol-3-yl)(3,4,5-trimethoxyphenyl)methanone) attenuated the expression of the most inflammatory factor mRNAs. RS-5645 also significantly reduced the cellular ratios of CD45+/IFNγ+, CD3+/IFNγ+, CD11b+/IFNγ+, and CD56+/IFNγ+ in human PBMCs. In addition, RS-5645 effectively inhibited the activation of inflammatory cells and reduced inflammatory damage to lung tissue in mice. Sequencing results of 16S rRNA v3+v4 in mouse alveolar lavage fluid showed that there were 494 OTUs overlapping between the alveolar lavage fluid of mice that underwent S protein+ LPS-combined intervention (M) and RS-5645-treated mice (R), while R manifested 64 unique OTUs and M exhibited 610 unique OTUs. In the alveoli of group R mice, the relative abundances of microorganisms belonging to *Porphyromonas*, *Rothia*, *Streptococcus*, and *Neisseria* increased significantly, while the relative abundances of microorganisms belonging to *Psychrobacter*, *Shimia*, and *Sporosarcina* were significantly diminished. The results of KEGG analysis indicated that the alveolar microbiota of mice in the R group can increase translation and reduce the activity of amino acid metabolism pathways. COG analysis results indicated that the abundance of proteins involved in ribosomal structure and biogenesis related to metabolism was augmented in the alveolar microbiota of the mice in the R group, while the abundance of proteins involved in secondary metabolite biosynthesis was significantly reduced. Therefore, our research results showed that RS-5645 attenuated pulmonary inflammatory cell infiltration and the inflammatory storm induced by the S protein and LPS by modulating the pulmonary microbiota.

## Introduction

Since the onset of the SARS epidemic, severe organ dysfunction and the extremely high fatality rate caused by cytokine storms (or inflammatory factor storms) have gradually attracted the attention of the medical community. After disease infections with H7N9 or H5N1 or with the recent corona virus disease 2019 (COVID-19), human hosts have shown varying degrees of cytokine storm syndrome 1, 2. Cytokines are a large class of multifunctional, small molecular weight, secretory proteins that are divided into two categories: one comprises the pro-inflammatory cytokines, which can facilitate the activation of many types of immune cells and promote the occurrence and development of inflammatory reactions 1, 3, 4. The second category entails the anti-inflammatory cytokines. Cytokines principally include interleukin (IL), interferon (IFN), chemokine (CK), tumor necrosis factor (TNF), the TGF-β family, colony-stimulating factor (CSF), and growth factors (GF). An increasing number of reports have indicated that inflammatory factor storms often occur in severe patients with novel corona virus pneumonia, but the mechanism appears complicated and the controls are limited 5. In pathologic sections from patients who died from complications of novel coronavirus infection, it was found that a large number of mononuclear macrophages infiltrated the alveoli, lung interstitium, and peri-pulmonary areas. With COVID-19, monocytes and macrophages also increased significantly, while peripheral blood CD4+ T cells decreased significantly, suggesting that there is a specific correlation between the dynamic changes in the number or function of host immune cells after viral infection and an inflammatory factor storm and prognosis 6-8.

The novel coronavirus severe acute respiratory syndrome coronavirus 2 (SARS-CoV-2) belongs to the β-coronavirus genus, which is characterized by an enveloped virus containing a large sense RNA genome wrapped in nucleocapsid protein 9-11. Three transmembrane proteins are integrated into the viral lipid envelope-i.e., spike (S) protein, a membrane protein, and an envelope protein 12, 13. The S protein trimer of SARS-CoV-2 binds to the ACE2 receptor on the surface of target cells and mediates the fusion of virus and cell, and the invasion and fusion of nucleic acid 13. Therefore, S protein is a very effective component of SARS-CoV-2 invasiveness. In a cohort study of 647 SARS-CoV-2 infected patients, investigators found that the antibody response strength and neutralizing antibody titers against S protein and nucleoprotein were closely related to clinical scores and prognosis 14. These results suggest that the S protein is also involved in the immune response of SARS-CoV-2-infected individuals. However, whether the S protein is involved in the inflammatory storm manifested within an infected person's body has not yet been reported.

3-Aroyl-1-arylpyrroles (ARAPs) and 3-aroyl-1,4-diarylpyrroles (ARDAPs) are potent inhibitors of tubulin assembly and growth of a panel of cancer cells by binding the colchicine site of tubulin 15-17. Further structure-activity relationship studies led to replace the 4-aminophenyl group at 4 position of the pyrrole nucleus with five- and six-membered bioisosteric heterocycles, while retaining the crucial methyl and fluoro substituents on the 1-phenyl ring.4 The compound RS5645 ((4-(thiophen-3-yl)-1-(*p*-tolyl)-1*H*-pyrrol-3-yl)(3,4,5-trimethoxyphenyl)methanone), designed as new potent inhibitor of tubulin polymerization, inhibited the proliferation of both glioblastoma multiforme and chronic myeloid leukemia cells, including those expressing the T315I mutation, at nanomolar concentrations^17^. *In vivo* experiments in BALB/c^nu/nu^ mice injected subcutaneously with U87MG cells, the same compound significantly inhibited tumor growth, tumorigenesis and angiogenesis. In addition, compound RS4645 was found to block human topoisomerase II (hTopoII) selectively and completely 17.

In view of the above evidence, in the present study we explored whether the SARS-CoV-2 (COVID-19) spike protein activates the body's immune system and causes a storm of inflammatory factors. We also wished to confirm whether RS-5645 inhibits the activation of immune cells and the occurrence of inflammatory storms, and to uncover its underlying molecular mechanism(s) of action.

## Materials and methods

### Isolation and treatment of peripheral blood mononuclear cells (PBMCs)

This experiment was carried out according to previously described methods 18. Briefly, 4 mL of human peripheral blood was collected from 4 healthy men each, and peripheral blood mononuclear cells (PBMCs) were isolated from the blood sample using the Cobe Spectra Apheresis System (CaridianBCT, USA). The PBMCs were treated with different concentrations of the 4-methylphenyl, thiophen-3-yl aroyl-diaryl-pyrrole (ARDAP) derivative referred to as RS-5645 and/or recombinant COVID-19 spike protein (S protein) for 24 h.

### Establishment of a mouse model and drug treatment

We herein applied methods previously published 17, 19. Ten-week-old male BALB/cByJ mice (n = 30) were purchased from the Experimental Animal Centre of Shanghai University of Traditional Chinese Medicine, and randomly allocated to 3 groups with 10 mice in each group. The blank control-group (UNTREATED) mice were fed with ordinary feed without any intervention. Mice in the treatment group (RS-5645) were intraperitoneally injected with 200 μL of mixed reagent containing 2.5-mg/kg RS-5645, 0.5-mg/kg lipopolysaccharide (LPS)^20^, and 10-mg/kg S protein every 2 d. The mice in the negative-control group (Saline) were intraperitoneally injected with 200 μL of mixed reagent containing 0.5-mg/kg LPS and 10-mg/kg S protein and an equal volume of saline every 2 d. Mice in each group received continuous treatment for 21 d. This study was approved by the Ethics Committee of the Shanghai Institute of Traditional Chinese Medicine and Gerontology (SHAGESYDW202006), and all experiments were in compliance with the Chinese National Science and Technology Commission's Laboratory Animal Regulations.

### Microbiota analysis of mouse bronchoalveolar lavage fluid

In brief, mouse bronchoalveolar lavage fluid samples were collected for microbial analysis as follows. Bacterial genomic DNA was extracted from frozen samples stored at -80°C. The V3 and V4 regions of the 16S rRNA gene were amplified by PCR using specific bacterial primers (F primer, 5'-ACTCCTACGGGAGGCAGCA-3'; R primer, 5'-GGACTACHVGGGTWTCTAAT-3'). High-throughput pyrosequencing of the PCR products was performed on an Illumina MiSeq platform at Biomarker Technologies Co., Ltd. (China). The raw paired-end reads from the original DNA fragments were merged using FLASH32 and assigned to each sample, according to the unique barcodes. The uclust tool of the QIIME software (version 1.8.0) was used based on 97% sequence identity, the tags were clustered into OTUs, and the alpha diversity index was evaluated using the Mothur software (version 1.30). To compare the diversity index among samples, we standardized the number of sequences contained in each sample. Analysis treasure included OTU rank, rarefaction, and Shannon curves; and the Shannon, Chao1, Simpson, and ACE indices were calculated. For beta diversity analysis, heatmaps of RDA-identified key OTUs, PcoA, NMDS, and UPGMA were obtained using QIIME. We used the LDA-effect size (LEfSe) method for the quantitative analysis of biomarkers in each group. We executed LEfSe analysis with an LDA threshold > 4, the non-parametric factorial Kruskal-Wallis sum-rank test, and the unpaired Wilcoxon rank-sum test to identify the most differentially abundant taxa.

### RNA extraction, reverse transcription, and quantitative real-time PCR (qPCR)

We followed the steps in the instructions of the RNAprep pure Tissue Kit (Tiangen Biotech Co., Ltd., Beijing, China), added 800 μl of lysate to the cells, and shook them vigorously. We then added 200 μl of chloroform, mixed the solution by inversion, centrifuged at 13,400×g at 4°C for 15 min, and removed the supernatant. We twice added an equivalent volume of absolute ethanol to the supernatant, mixed by inversion, and centrifuged at 13,400×g for 30 min at 4°C. The RNA pellet was then resuspended in 500 μl of 75% ethanol, centrifuged at 13,400×g for 5 min at 4°C. We removed excess liquid and added 300 μl of DECP water to the precipitate to fully dissolve it. We took 1 μl of RNA solution to calculate the ratio of OD260/OD280 (generally controlled between 1.8 and 2.0) to determine the purity and total concentration of RNA. Following the steps in the messenger RNA (mRNA) first-strand complementary DNA (cDNA) (Tiangen Biotech Co., Ltd., Beijing, China) manual, we took 20 μl (100 ng/μl) of total RNA, a 2×mRNA RT reaction buffer of 25 μl, 1×mRNA RT enzyme mix of 4 μl, and 6 μl of RNase-free deionized water and mixed them thoroughly. The following reactions were carried out in a PCR machine: initially at 42°C for 60 min, we performed an miRNA-poly A tailing reaction and reverse-transcription reaction; and then at 95°C for 3 min, we followed with an enzyme-inactivation reaction. According to the steps of the mRNA qPCR Detection kit (Tiangen Biotech Co., Ltd., Beijing, China) instructions, we added reagents, test samples, and primers in the following sequence: 10 μl of 2 × mRcute PlusmRNAPremix (with SYBR), 1 μl of 1 × forward primer and reverse primer each (10 μM), 4 μl of mRNA first-strand cDNA, and 4 μl of deionized water. Using a real-time fluorescence quantitative PCR machine, we performed the following reactions: 95°C for 15 min, 94°C for 20 s, and 60°C for 34 s; and we then read the fluorescence value. The above reaction was performed for 40 cycles. We used the 2^-ΔΔCt^ calculation method to determine the relative gene expression, where ΔCt = Ct_genes-Ct_18s RNA, and ΔΔCt = ΔCt_all_groups-ΔCt_control_group. The mRNA expression level was then corrected according to the expression level of 18s rRNA.

### Western immunoblotting analysis

The total protein of each group of cells was used for 12% SDS-PAGE denaturing gel electrophoresis, and after completion it was transferred to a PVDF membrane (Millipore). After blocking and washing the membrane, we incubated it with primary antibodies at 37°C for 45 min; and after washing the membrane thoroughly, we incubated with second antibodies at 37°C for 45 min. We washed the membrane with TBST 4 times at room temperature for 14 min each time, and then exposed and developed it (Sigma-Aldrich Chemical) using the ECL-enhanced chemiluminescence method (ECL kit, Pierce Biotechnology).

### Hematoxylin-eosin staining (H&E staining)

The tissue samples were fixed in 4% paraformaldehyde, dehydrated, embedded in paraffin, sectioned at 4 μm on a paraffin microtome, and placed on a glass slide. Subsequently, we dewaxed the sections with xylene and immersed them in a graded series of ethanol (100%, 90%, 80%). We stained with hematoxylin staining solution at room temperature for 5 min; 1% hydrochloric acid ethanol was used for differentiation for 30 s, light ammonia water was added to turn the sections blue for 1 min, and we rinsed them with distilled water for 5 min. We subsequently stained the sections with eosin at room temperature for 2 min, and washed the slides with distilled water for 2 min. We then dehydrated the sections in a graded series of ethanol (75%, 80%, 95%, 100%) and allowed decolorization. We permeated cells with xylene for 2 min and ultimately sealed the sheet with neutral gum.

### MASSON staining

According to our previous study 21, sections were washed with double-distilled water for 5 min and then stained with hematoxylin (Beyotime Biotechnology) for 5-10 min, followed by thorough rinses with water. The sections were subsequently counterstained with Masson's Ponceau Acid Fuchsin solution (Beyotime Biotechnology) for 6-10 min, and then rinsed in 2% ice-cold aqueous acetic acid (Beyotime Biotechnology) for 5 s. We differentiated the sections for 3-5 min with 1% aqueous phosphomolybdic acid (Beyotime Biotechnology), stained them by direct immersion in aniline blue for 5 min, and then washed them with 0.2% aqueous glacial acetic acid (Beyotime Biotechnology) for several seconds. The stained sections were cleared, sealed, and photographed.

### Immunohistochemical staining

Briefly, all fresh tissues were immersed in 4% paraformaldehyde (Sigma-Aldrich, St. Louis, USA) for 30 min at room temperature; and we performed ethanol-gradient dehydration, paraffin embedding, sectioning at 6 μm, and dewaxing in xylene. Tissue sections were blocked with immunohistochemical blocking solution (Beyotime Biotechnology Co., Ltd., Zhejiang, China) at 37°C for 30 min. We discarded the blocking solution, added the immunohistochemical cleaning solution (Beyotime Biotechnology Co., Ltd., Zhejiang, China), and washed the sections three times at room temperature for 5 min each. The primary antibody was added and the sections incubated at 37°C for 45 min. We discarded the antibody, added the immunohistochemical cleaning solution (Beyotime Biotechnology Co., Ltd., Zhejiang, China), and washed the sections at room temperature three times for 5 min each. The secondary antibody was then added, and sections were incubated at 37°C for 45 min. The antibody was discarded, we added the immunohistochemical cleaning solution (Beyotime Biotechnology Co., Ltd., Zhejiang, China), and then washed the sections at room temperature three times for 5 min each. Finally, we added the immunofluorescence blocking solution (Sigma-Aldrich, St. Louis, USA) for mounting.

### Flow-cytometric analysis

After centrifugation of each group of cells, the pellet was collected, and the cell density was adjusted to 1×10^6^ cells/ml with Dulbecco's phosphate-buffered saline (DPBS) containing 10% BSA, and the total volume was maintained at 0.2 ml. We added primary antibodies to the cell suspension, mixed them well, and allowed them to react for 30 min in the dark at 4ºC. Cell staining was compared with an isotype control antibody (mouse IgG1-FITC, mouse IgG1-PE Invitrogen, eBioscience, Shanghai, China) to correct for non-specific binding. Evaluation of antibody staining by flow cytometry (FCM) was performed using a FACS Aria (Quanta SC, Beckman Coulter, Inc.).

### Statistical analysis

Each experiment was performed as least three times, and data are shown as the mean+/-standard error, where applicable. Differences were evaluated with Student's *t*-test. A P value less than 0.05 was considered statistically significant.

## Results

### RS-5645 effectively inhibits inflammatory cell activation and inflammatory factor storm induced by the COVID-19 spike protein

**Figure 1:**
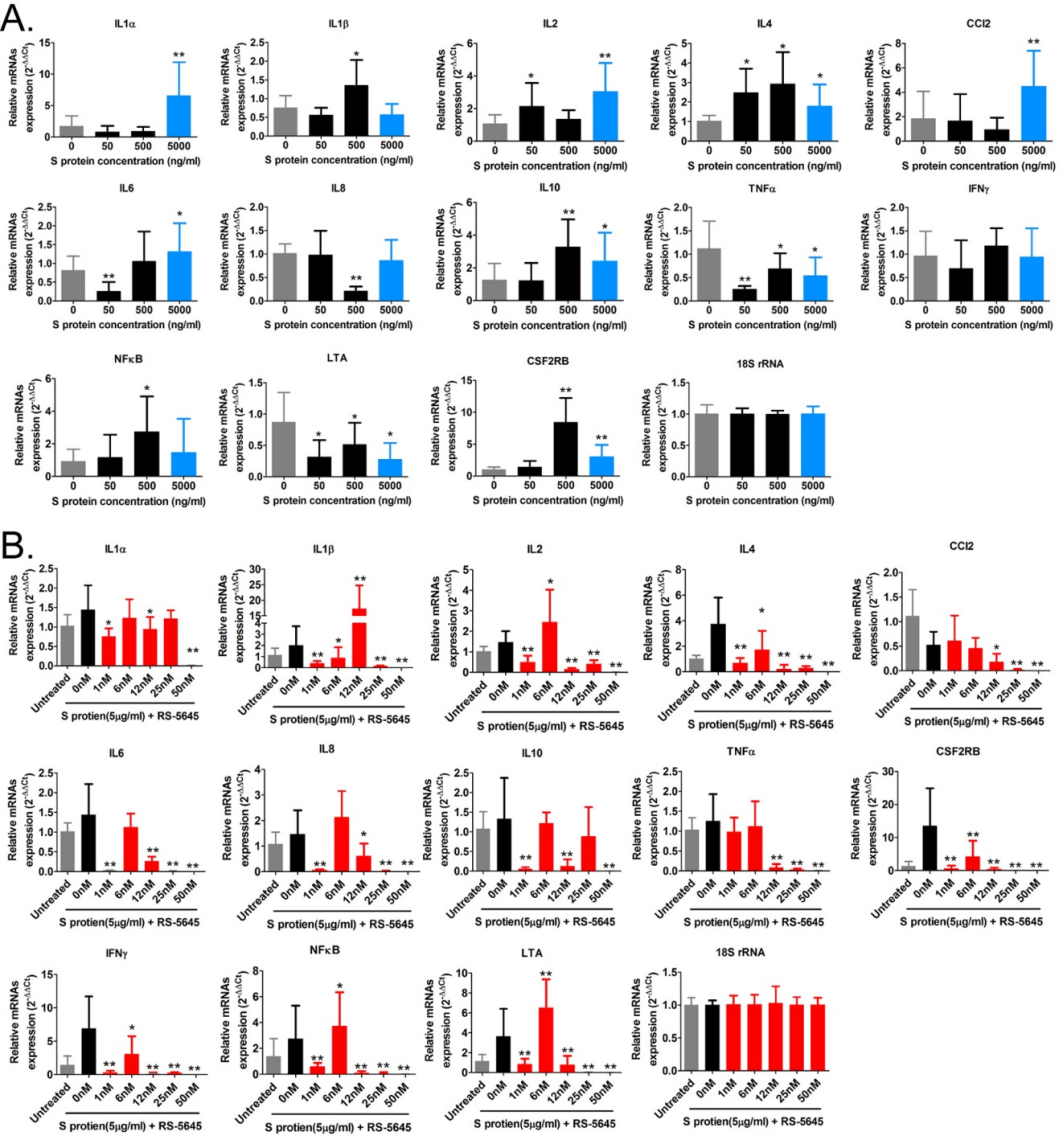
The effect of RS-5645 on the activation of human PBMCs and the release of inflammatory factors induced by the S protein. (A) qPCR results indicated that the S protein induced the activation of human PBMCs and the upregulation of inflammatory factor gene mRNA expression. **p < 0.01 vs. 0 ng/ml; *p < 0.05 vs. 0 ng/ml; *t*-test; n = 3. (B) qPCR results indicated that RS-5645 significantly inhibited human PBMC activation and inflammatory factor gene mRNA expression. **p < 0.01 vs. 0 nM; *p < 0.05 vs. 0 nM; *t*-test; n = 3.

**Figure 2:**
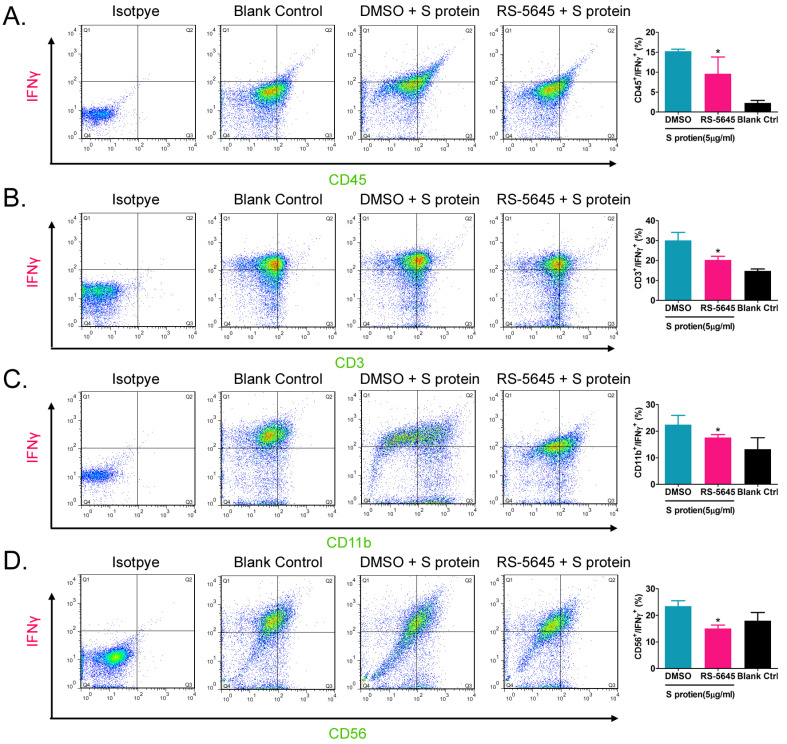
Flow-cytometric results. (A) The ratios of CD45+/IFNγ+ cells among different groups. *p < 0.05 vs. DMSO group; *t*-test; n = 3. (B) The ratios of CD3+/IFNγ+ cells among different groups. *p < 0.05 vs. DMSO group; *t*-test; n = 3. (C) The ratios of CD11b+/IFNγ+ cells among different groups. *p < 0.05 vs. DMSO group; *t*-test; n = 3. (D) The ratios of CD56+/IFNγ+ cells among different groups. *p < 0.05 vs. DMSO group; *t*-test; n = 3.

In order to determine whether the COVID-19 spike protein can activate mononuclear cells and induce secretion of inflammatory factors, we used 3 different concentrations (50, 500, and 5000 ng/ml) of S protein to stimulate human primary PBMCs. Our qPCR results indicated that 50 ng/ml of S protein activated the expression of some inflammatory factor genes (Figure 1A). As the concentration of S protein increased, the mRNA expression levels of multiple inflammatory factors commensurately and significantly increased compared to the control group. (0 ng/ml). Our experimental results suggested that the COVID-19 spike protein activated PBMCs to release inflammatory factors. Subsequently, we tested whether different concentrations of RS-5645 could effectively inhibit the COVID-19 spike protein (up to 5000 ng/ml)-induced PBMC activation and inflammatory factor storm. qPCR results showed that different concentrations of RS-5645 inhibited the expression of some inflammatory factor gene mRNAs (Figure 1B), and when the RS-5645 concentration reached 25 or 50 nM, the expression levels of almost all of the inflammatory factor gene mRNAs were significantly downregulated compared with the control group (0 nM). In addition, the flow cytometric results revealed that white blood cells (CD45+/IFNγ+), T cells (CD3+/IFNγ+), monocytes (CD11b+/IFNγ+), and NK cells (CD56+/IFNγ) in human PBMCs that were altered by S protein were significantly higher than in the control cells (Figure 2A-D), suggesting that a large number of mononuclear cells were activated. However, after RS-5645 treatment, the ratios of CD45+/IFNγ+, CD3+/IFNγ+, CD11b+/IFNγ+, and CD56+/IFNγ+ were lower than those for the model group. We surmised that RS-5645 could effectively inhibit inflammatory cell activation and inflammatory factor storms induced by the COVID-19 spike protein.

### RS-5645 effectively inhibits the activation of inflammatory cells and inflammatory damage to lung tissue in mice

**Figure 3:**
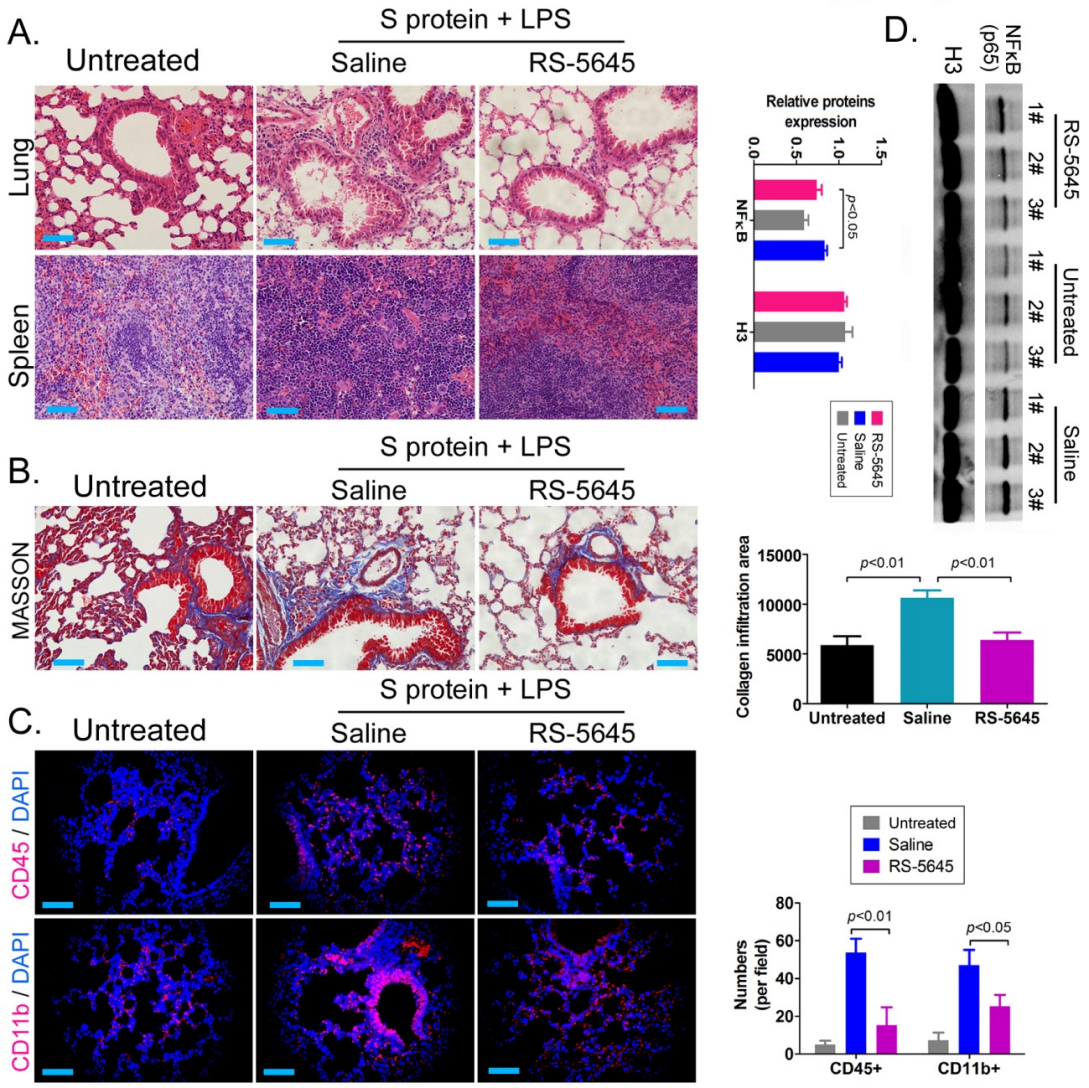
RDS-5645 reduces lung damage in mice caused by inflammatory factors. (A) H&E staining results of mouse lungs and spleen (x400, scale bar = 30μm). (B) MASSON staining results of mouse lungs (x400, scale bar = 30μm). (C) Immunofluorescence staining results of mouse lungs (x400, scale bar = 30μm). (D) Western blot analysis of nuclear protein expression levels of PBMCs in each group of mice.

In order to explore whether RS-5645 also effectively inhibited the activation of inflammatory cells in vivo, we used the COVID-19 spike protein, combined with LPS, to construct a mouse model of subacute inflammatory factor storms and then intervened using RS-5645. The H&E staining results showed that in the lung tissue of the mouse model characterized by S protein+LPS-combined treatment, we uncovered multiple mononuclear cell infiltrations in the bronchi and alveolar cavities, accompanied by diffuse congestion or hemorrhagic necrosis in some lung tissues and congestion of lung sections. We observed signs of changes in bleeding, small blood vessel proliferation, blood vessel wall thickening, and luminal stenosis and occlusion. However, the lung tissue damage in the RS-5645 treatment group mice was significantly less than that in the model-group mice (Figure 3A). The splenic monocytes in the model group also increased significantly with megakaryocytes, while the splenic injury in the RS-5645-intervention group significantly decreased (Figure 3B). The results of MASSON staining indicated that RS-5645 intervention significantly reduced pulmonary interstitial fibrosis with partial hyaline degeneration in the mice (Figure 3C). IF staining in the lungs of the model group exhibited interstitial infiltration of multiple inflammatory cells, including leukocytes (CD45+) and monocytes (CD11b+) (Figure 3D). However, RS-5645 significantly reduced the lung infiltration of inflammatory cells in the mice. In addition, western blotting analysis showed that the expression level of NFκB protein in PBMC nuclei of the RS-5645 intervention group was significantly lower than that in the model group (Figure 3D). We concluded that RS-5645 effectively inhibited the activation of inflammatory cells and inflammatory damage to lung tissue in mice.

### RS-5645 significantly modulates the lung microecology of S protein+LPS- combined intervention in the mouse model

**Figure 4:**
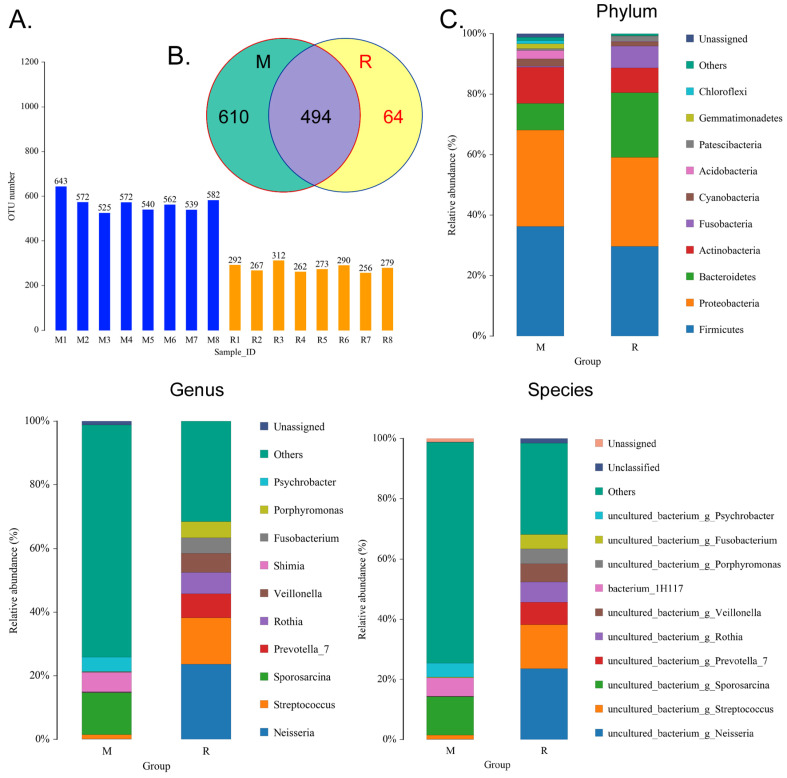
Analysis of operational taxonomic units (OTAs). (A) Number of OTAs. (B) Venn diagram of OTAs. (C) Pulmonary microbiota clustering and species distribution.

**Figure 5:**
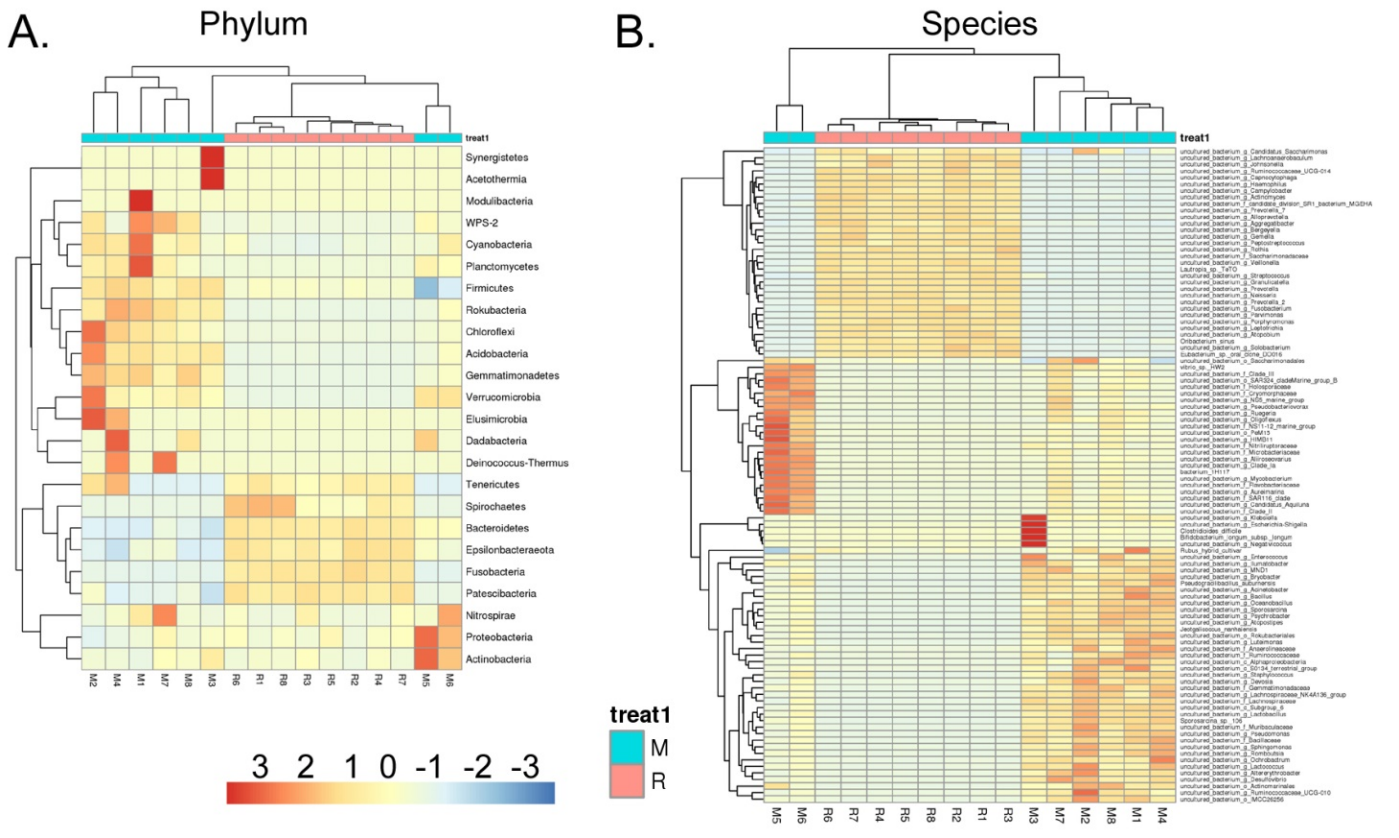
Heatmap of species-richness clustering at the level of (A) phylum, (B) species.

**Figure 6:**
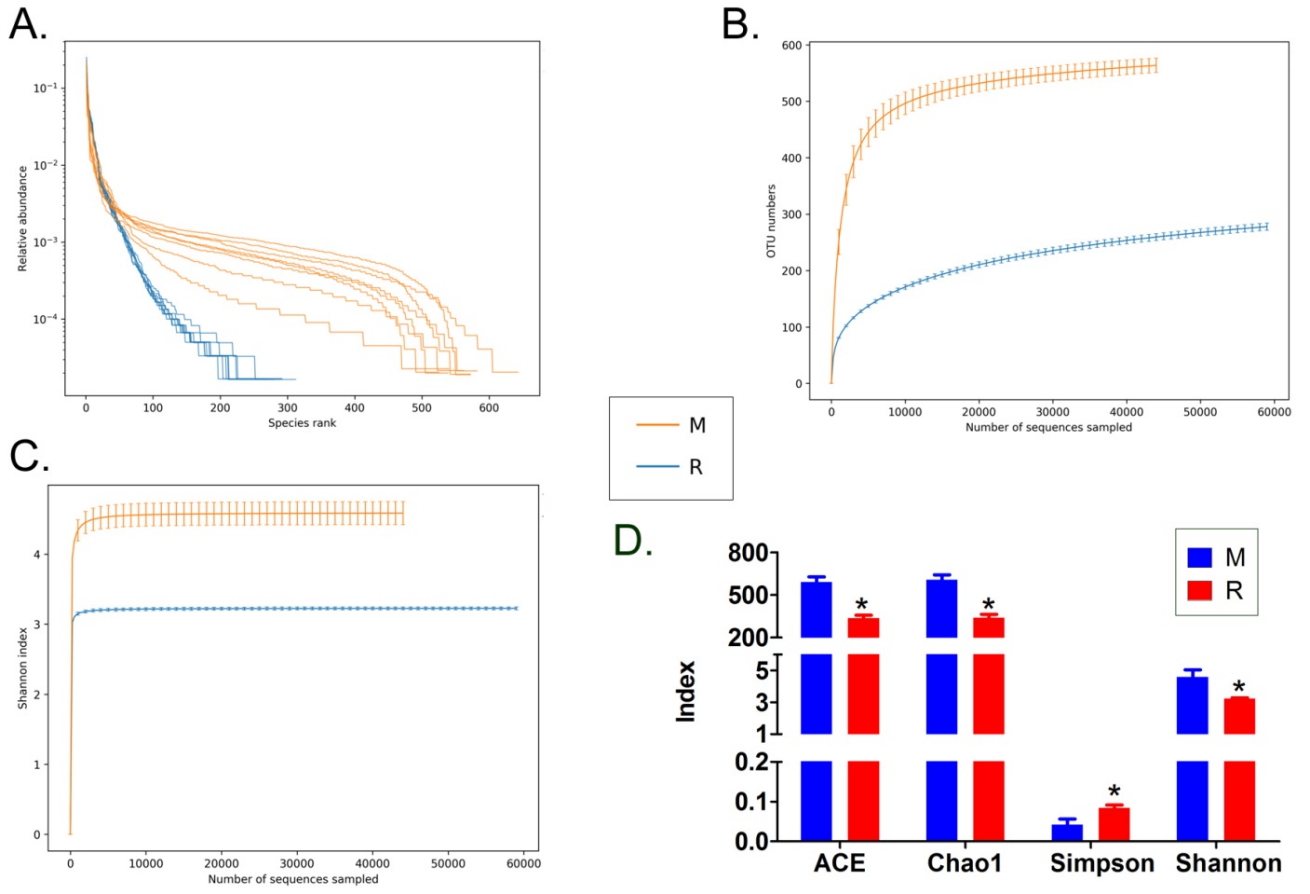
Analysis of alpha diversity. (A) Rank abundance curve, (B) rarefaction curve, (C) Shannon index curve, (D) alpha-diversity analysis.

**Figure 7:**
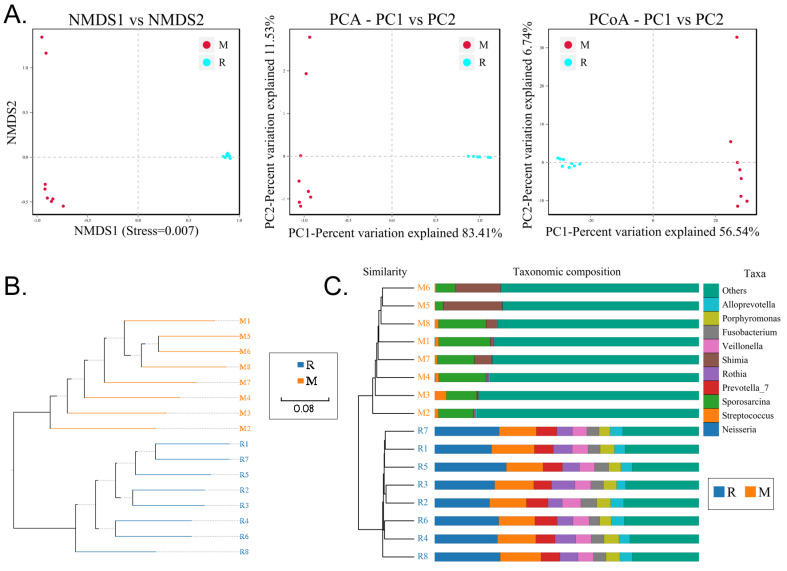
Analysis of beta diversity. (A) Results of principal component analysis, principal coordinate analysis, and non-metric multidimensional scaling analysis. (B) Unweighted-sample, pair-group method with arithmetic-mean clustering tree. (C) Combined illustration of clustering tree and histogram.

**Figure 8:**
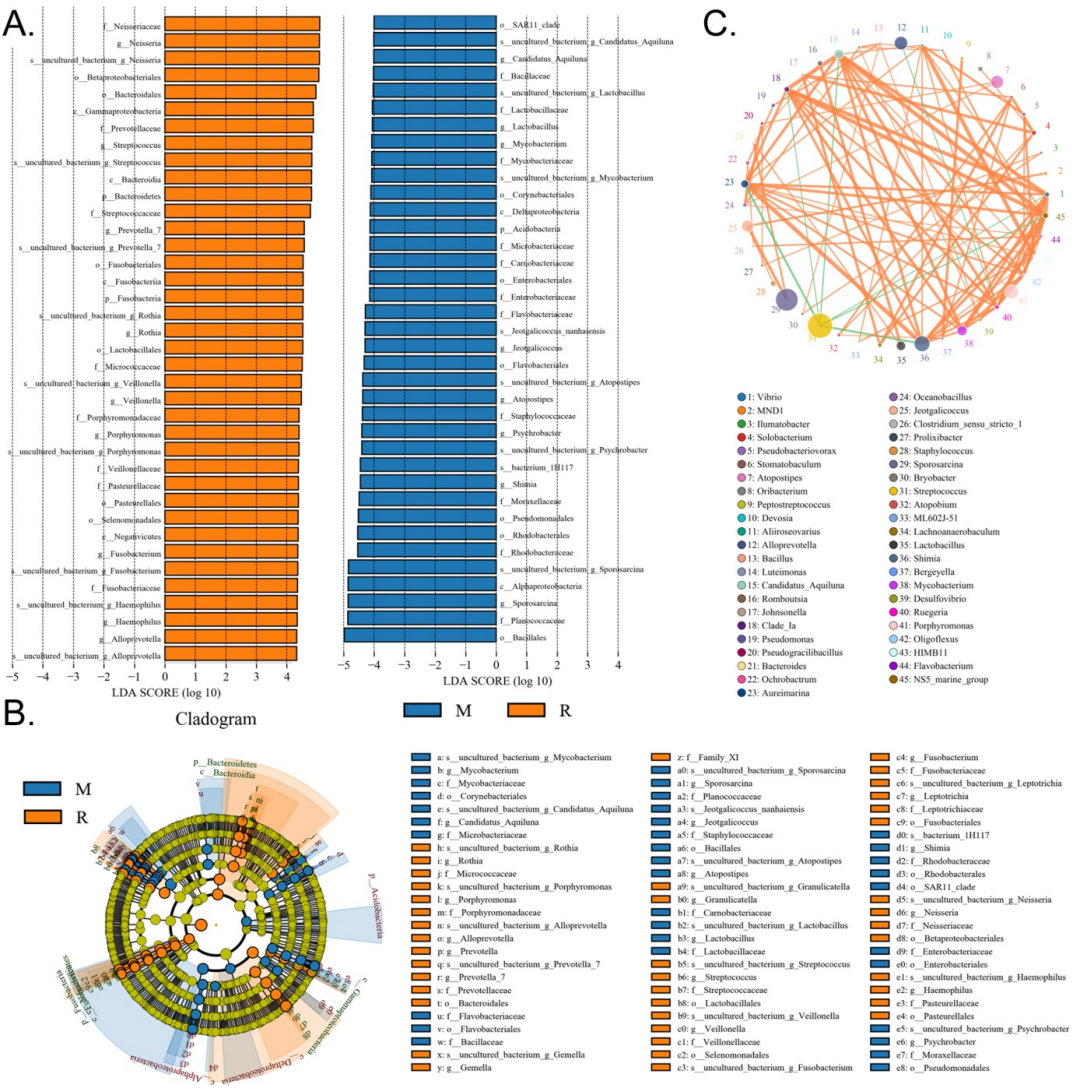
Significant-difference analysis between groups. (A) Value-distribution histogram of line discriminant analysis effect size (LEfSe). (B) Results of species annotation were visualized using KRONA. (C) Species network at the genus level.

We collected the alveolar lavage fluid (R) from the S protein+LPS-combined-intervention mouse model with RS-5645 treatment and the alveolar lavage fluid (M) of the control-group mice receiving only saline. We then sequenced the bacterial 16S rRNA v3+v4 region to assess the composition of the lung microbiota and the distribution of specific flora. A total of 1,278,766 pairs of reads were obtained by sequencing 16 samples, and a total of 1,243,873 clean tags were generated after splicing and filtering of double-ended reads. Each sample generated at least 77,123 clean tags, with an average of 77,742 clean tags. We used the uclust tool of the QIIME software (version 1.8.0) to cluster the tags into operational taxonomic units (OTU) based on 97% sequence identity. There was a significant difference in the number of OTUs between the R and M groups (Figure 4A); there were 494 OTUs overlapping between the two, while R had 64 unique OTUs and M had 610 unique OTUs (Figure 4B). By comparing the representative sequence of OTUs with the microbial reference database, each OTU could be divided into species, and we calculated the community composition of each sample. At different taxonomic levels (kingdom, phylum, class, order, family, genus, and species), we used QIIME software to generate species abundance tables of different taxonomic levels. The community structure of samples with different classification levels was drawn using R language tools (Table S1). Phylum-level analysis showed that compared with the control group M, the relative abundances of microorganisms belonging to Bacteroidetes and *Fusobacteria* increased significantly in the alveoli of mice in the R group, while the relative abundances of microorganisms belonging to Actinobacteria, Firmicutes, and Acidobacteria decreased significantly. Genus-level analysis showed that compared with the control group M, the relative abundances of microorganisms belonging to *Porphyromonas, Rothia, Streptococcus*, and *Neisseria* increased significantly in the alveoli of mice in R group, while the relative abundances of microorganisms belonging to *Psychrobacter, Shimia, and Sporosarcina* decreased significantly. Species-level analysis showed that compared with the control group M, the relative abundances of microorganisms belonging to uncultured_bacterium_g_*Fusobacterium*, uncultured_bacterium_g_*Porphyromonas*, uncultured_bacterium_g_*Rothia*, uncultured_bacterium_g_*Streptococcus*, uncultured_bacterium_g_cultured_bacterium_g_*Neisseria*, and the relative abundances of microorganisms belonging to uncultured_bacterium_bacterium_gun_*Neisseria_and Porphyromonas*; and the relative abundances of microorganisms belonging to uncultured_bacterium_g_*Fusobacterium*, uncultured_bacterium_g_*Porphyromonas*, uncultured_bacterium_g_*Streptococcus*, and uncultured_bacterium_g_*Neisseria_bacterium* declined (Figure 4C). The results of cluster analysis of microbial diversity showed that most of the alveolar microbial diversity of mice in the R group came from Spirochaetes, Bacteroidetes, Epsilonbacteraeota, *Fusobacteria*, Patescribacteria, and other microorganisms (which increased in number), as well as from WPS-2, Cyanobacteria, Planctomycetes, Rokubacteria, Chloroflexi, Acidobacteria, Gemmatimonadetes, Verrucomicrobia, and other microorganisms (which decreased in number) (Figure 5). The α diversity analysis found that the rank abundance curve was flat, indicating a higher degree of uniformity in species composition (Figure 6A, Table S2); the Shannon index tended to be flat, indicating that the amount of sequencing data was large enough; and the OTU types did not increase with the increase in sequencing volume. Regarding growth (Figure 6B), the rarefaction curve was smooth, indicating that the sample sequence was sufficient for data analysis (Figure 6C). The β diversity analysis revealed that the Bray-Curtis algorithm could be used to analyze the differences between groups of microbial flora. The analysis primarily included principal coordinate analysis (PCOA), principal component analysis (PCA), and non-metric multi-dimensional scaling (NMDS). The above analysis showed that the microbial communities of the R and M groups were obviously different in the distribution of group owners; i.e., the R group and M group microorganisms formed separate communities (Figure 7A). The unweighted pair-group method with arithmetic mean (UPGMA) sample hierarchical cluster analysis suggested that the alveolar microbiota of group R and M had low homology, and that there was no close genetic background (Figure 7B, C). In addition, we used the line discriminant analysis (LDA) effect size (LEfSe) method to identify high-dimensional biomarkers in each group of intestinal microbiota. The LDA score was set to 4.0, and LDA scores greater than 4 for different species were considered to be important biomarkers. As shown by the cladogram analysis and LDA score distributions, for the R group the number of microorganisms in the g__*Neisseria,* f__Neisseriaceae, s__uncultured_bacterium_g_*Neisseria*, o__Bacteroidales, f__Prevotellaceae, g_*_Streptococcus*, and other families increased significantly. Therefore, the aforementioned microbial community was the unique dominant species of the R group (Figure 8A-8C).

### RS-5645 significantly regulates the differential expression of the S protein+LPS-combined intervention of 16S functional genes and metabolic signaling pathways in the lungs of mouse models

**Figure 9:**
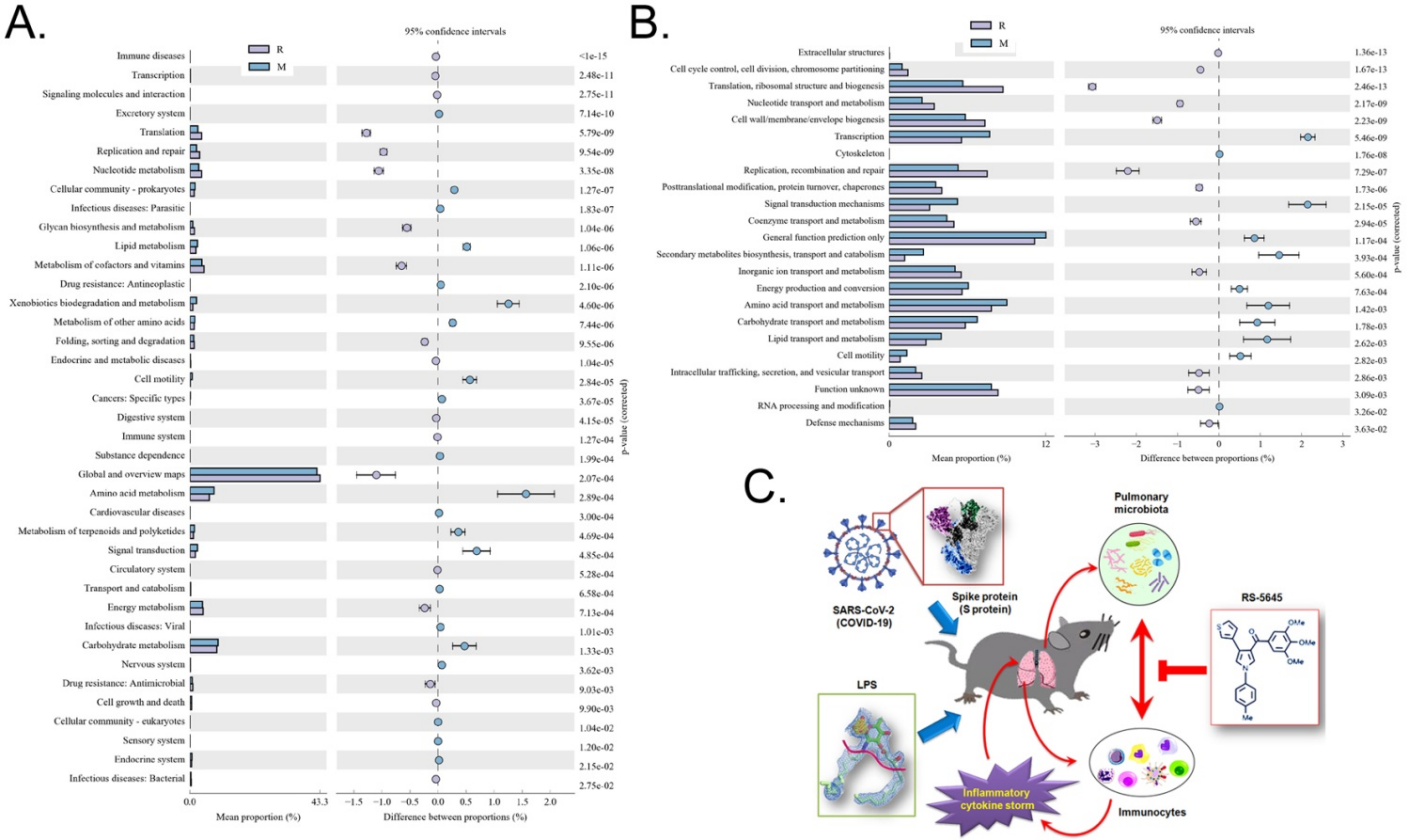
Metabolic-signaling pathways and protein differences in pulmonary microbiota. (A) Results of Kyoto Encyclopedia of Genes and Genomes (KEGG) metabolic pathway analysis. (B) Clusters of orthologous groups following protein analysis of distributions and abundances of homologous protein clusters in pulmonary microbiota. (C) Mechanism underlying the attenuation by RS-5645 of pulmonary inflammatory cell infiltration and inflammatory storms induced by the SARS-CoV-2 (COVID-19) spike protein and LPS is shown to occur via modulation of the pulmonary microbiota.

Through the differential analysis of KEGG metabolic pathways it is possible to observe the differences and changes in the metabolic pathways of the functional genes of the microbial community between samples of different groups, so as to explore the changes in metabolic functions of different samples in their adaptation to environmental changes (Table S3). The analysis showed that compared with the M group, the alveolar microbiota of mice in the R group increased in the metabolic pathways of translation (Genetic Information Processing), global and overview maps (Metabolism), and nucleotide metabolism (Metabolism), while it decreased in amino acid metabolism, metabolism (Metabolism), xenobiotic biodegradation and metabolism (Metabolism), and other substances (Figure 9A). Through clusters of orthologous groups of proteins (COG) analysis, the distribution and abundance of homologous protein clusters in the microbial community can be found (Table S4). Our results showed that compared with the M group, the mouse alveolar microbiota and metabolism-related translation, ribosomal structure and biogenesis (Information Storage and Processing), replication, recombination and repair (Information Storage and Processing), and Cell wall/membrane/Envelope biogenesis (Cellular Processes and Signaling) protein abundance all significantly increased, while metabolism-related transcription (Information Storage and Processing), signal transduction mechanisms (Cellular Processes and Signaling), secondary metabolite biosynthesis, transport and catabolism (Metabolism), amino acid, protein abundance of transport and metabolism (Metabolism), and lipid transport and metabolism (Metabolism) all significantly decreased (Figure 9B).

## Discussion

In the present study, we explored three questions in detail. First, does S Protein cause a storm of inflammatory factors by activating PBMCs? Second, does the combination of S protein and low-dose LPS cause lung inflammation in mice? Third, does the anti-tumor drug RS-5645 inhibit the inflammatory storm.

According to several research reports, the histopathology of the lungs of patients who died of SARS-CoV-2 (COVID-19) showed a large amount of pulmonary interstitial fibrosis with partial hyaline degeneration and pulmonary hemorrhagic infarction 22-29; proliferation of small blood vessels, thickening of blood vessel walls, stenosis and occlusion of the lumen; and interstitial infiltration of inflammatory cells, including lymphocytes, plasma cells, and monocytes 22-29. Alveolar inflammation is accompanied by various changes in alveolar epithelial cells (principally type II), such as atrophy, proliferation, desquamation, and squamous metaplasia 22-29. We therefore used the COVID-19 spike protein combined with LPS to construct a subacute inflammatory-storm mouse model. The pathologic diagnostic results of the mouse lungs also showed some of the above-mentioned pathologic manifestations-particularly pulmonary interstitial fibrosis, pulmonary hemorrhagic infarction, blood vessel wall thickening, stenosis and occlusion of the lumen, and a large amount of infiltration into the inflammatory cell interstitium. We thus demonstrated that the pathologic characteristics of the mouse model constructed using this method were very similar to the human lungs after COVID-19 infection. This model provides a plausible way to study the pathogenic mechanism of COVID-19, and to seek effective therapeutic targets in the future. In addition, many reports have confirmed that COVID-19 and SARS-CoV utilize the same cell-entry receptor (i.e., ACE2 [angiotensin-converting enzyme II). Under normal circumstances, ACE2 protein is expressed in alveolar cells, bronchial epithelium, and vascular endothelial cells; and the COVID-19 spike protein has been shown to bind to ACE2 to promote the introduction of viral nucleic acid into host cells, which in turn leads to acute lung injury and pulmonary edema. However, it noteworthy that we used the COVID-19 spike protein to directly interfere with human PBMCs, which can trigger their activation and the expression of multiple inflammatory factor genes. Although we have not studied the underlying mechanism(s) in depth, we speculate that there may be corresponding S protein receptors on some subgroups of PBMC cells-or even the presence of ACE2 itself. However, this hypothesis still needs to be verified through detailed experiments in the future. Herein we postulated that the cause of lung injury in the mouse model was precisely the result of S protein combined with LPS. Cytokine storms are related to an excessive immune response and an uncontrolled pro-inflammatory response, with the latter leading to serious organ diseases-including lung damage. Several representative cytokines have been identified-including IL-1β, IL-18, TNF-α, IL-6, IL-8, and IL-10-which are produced by various immune cells that include CD8 and CD4. In our hands, LPS activated mononuclear cells and induced the release of multiple inflammatory factors. COVID-19 spike protein also effected acute lung injury and pulmonary edema by binding to the ACE2 receptor of alveolar cells.

The cytokine or inflammatory storm-also known as the cytokine cascade, or hypercytokinemia-was first proposed by Ferrara et al. in 1993 in graft-versus-host disease (GVHD). It is caused by infection, drugs, or certain diseases that eventuate in excessive immunity within the body 30. If the mild and secondary organ dysfunction during the inflammatory response assists the host in overcoming the infection and survive, then the inflammatory response is evolutionarily acceptable; i.e., the body's defense and repair responses are positive 1, 3, 4, 30. If the inflammatory response leads to excessive organ dysfunction-i.e., putting the host at risk in terms of survival and reproduction-then this latter inflammatory response is pathologic 1, 3, 4, 30. There are extensive regulatory mechanisms present in our bodies that modulate immune responses and prevent cytokine storms. In the case of severe infections or multiple-pathogen infections due to the host's own low immune-regulatory ability, cytokine storms continue to occur and accumulate in systemic organs, causing multiple organ-system failure. After severe infection, the boundary between a normal response and unregulated response becomes blurred as certain cytokines may not only help control infection, but also be harmful to the host. The interdependence of these inflammatory mediators further complicates the distinction between normal and dysregulated responses 1, 3, 4, 30. Similarly, the generation of a cytokine storm is not only related to the pathogen's own stimulation of the host, but also to the host's own immune-regulatory capability and to the ecologic microenvironment of specific tissues and organs. These factors certainly show complexity and diversity 1, 3, 4, 30. In this study, we confirmed that the S protein from the pathogen SARS-CoV-2 activated human PBMC cells and induced inflammatory storms. This confirms that-in addition to its own separate effects on patients with severe COVID-19-the SARS-CoV-2 pathogen protein itself constitutes one of the important factors that can create an inflammatory factor storm in an infected individual.

Conversely, changes in the local microecology of tissues and organs will also aggravate the degree of organ damage and the occurrence of chronic inflammation. It is increasingly recognized that microorganisms directly or indirectly regulate tolerance and control the process of inflammation. The abundances and functional or anatomical positioning of the microbiotas can lead to the loss of immune tolerance, which in turn leads to the development of inflammation and immune-mediated pathology 31-33. Under immune homeostasis, the immune system can tolerate beneficial commensal bacteria, but will also respond to these same microbiota during periods of tissue rupture or other homeostatic disturbances 31. Investigators have found that after exposure to house dust mite allergens, newborn mice exhibit increased airway eosinophil numbers and undergo release of TH2 cytokines, which then weaken with the increase in bacterial load in the lungs 31. Colonization of microbial flora in newborn mice can reverse allergic reactions, but not in adult mice 31. The aforementioned studies indicate that the lung microbiota has a certain regulatory effect on allergen-induced immune activation. The lifetime development of the human upper respiratory tract microbiota appears to be negatively or inversely correlated with susceptibility to community-acquired pneumonia 34. By-products of lung inflammation (such as nitrates)-particularly in COPD patients-can be used by some gram-negative bacteria to grow and surpass members of the normal microbiota 34. The numbers of *Pseudomonas, Haemophilus, Moraxella,* and *Streptococcus spp.* in the lower respiratory tract of patients with pneumonia thus increase, and the increased colonization of opportunistic pathogens in patients with chronic lung disease may lead to an increased risk for lower respiratory tract infections 34. A large number of *Haemophilus* bacteria were also found in the lower respiratory tract of asthma patients 34. In addition, peptidoglycan fragments produced by the respiratory microbiota stimulate alveolar macrophages mediated by NOD2 receptors, IL-17, and granulocyte-macrophage colony stimulating factor (G-MCSF) in order to kill pathogens. Short-chain fatty acids (SCFAs) can also enhance the antibacterial activity of mouse neutrophils and macrophages. Although the microbiota is involved in limiting the production of IL-10, thereby enhancing the neutrophil-dependent lung defenses of mice against *Klebsiella pneumonia,* microbial SCFAs have also been contrarily shown to promote the production of IL-10 34. All of the above evidence shows that lung (respiratory tract) microorganisms are closely related to pulmonary inflammation and cytokine production. Our present research therefore confirmed that the relative abundances of *Porphyromonas, Rothia, Streptococcus,* and *Neisseria* microorganisms increased significantly with RS-5645 treatment in mouse alveolar lavage fluid, while the relative abundances of *Psychrobacter, Shimia, and Sporosarcina* microorganisms decreased significantly. Studies have revealed that microorganisms of the genus *Sporosarcina* can usually be found in bronchial biopsies of children with cystic fibrosis 35. The Nicod team also reported that *Porphyromonas, Rothia, Streptococcus*, *Neisseria,* and other microorganisms are the principal constitutive members in the lungs of healthy individuals 36. It can be seen that our research results are basically consistent with existing reports that indicate that RS-5645 not only alleviates the organ damage caused by inflammatory-factor storms, but also regulates the microbial flora of the lungs, allows pathogenic flora to revert to normalized flora, and promotes organ repair and micro-ecologic balance. In addition, according to our study, RS-5645 attenuated murine inflammatory cytokine storm and lung inflammation induced by SARS-CoV-2 spike protein and LPS by targeting multiple targets. In this study, we found that LPS and SARS-CoV-2 spike protein and LPS induced systemic inflammation and lung inflammatory injury in mice were closely related to the imbalance of pulmonary microbiota, inflammatory cell infiltration, signal transduction pathway of inflammatory factor activation, and metabolites of microbiota. Therefore, the effects of the above factors on the host cannot be ignored.

In summary, our research revealed that the spike protein of SARS-CoV-2 induced a storm of inflammatory factors by activating PBMC cells, and that RS-5645 significantly inhibited immune cell activation and inflammatory cytokine storms. RS-5645 also promoted the transformation of pathogenic bacteria in the lungs of mice to more normalized flora, rebuilt the micro-ecologic balance, and executed two actions cooperatively to ultimately ameliorate the inflammatory damage to the lungs.

## Supplementary Material

Supplementary tables.
