# Supplementary material for: RS-5645 attenuates inflammatory cytokine storm induced by SARS-CoV-2 spike protein and LPS by modulating pulmonary microbiota
Source: Int J Biol Sci. 2021 Jul 25;17(13):3305–19. doi: 10.7150/ijbs.63329 (PMC8416739; doi:10.7150/ijbs.63329)
Supplement: Supplementary file 1 — Supplementary tables. [file ijbsv17p3305s1.pdf]

Table S1 Statistical table of per level species of samples

Sample	Kindom	Phylum	Class	Order	Family	Genus	Species
M1	1	17	46	122	203	364	390
M2	1	17	47	116	195	341	363
M3	1	16	42	104	182	319	339
M4	1	19	45	113	198	337	358
M5	1	17	40	107	189	322	344
M6	1	16	39	107	191	341	362
M7	1	17	41	115	195	326	348
M8	1	17	42	110	198	336	359
R1	1	18	35	63	103	188	196
R2	1	16	30	61	94	168	174
R3	1	16	34	70	109	184	191
R4	1	16	31	59	94	161	169
R5	1	16	31	60	93	172	177
R6	1	16	31	62	100	185	192
R7	1	16	31	58	92	162	168
R8	1	18	36	69	101	169	174
Total	1	24	63	165	286	563	610

Table S2 Alpha diversity index statistics

Sample ID	OTU	ACE	Chao1	Simpson	Shannon	Coverage
M1	643	670.847	675.2174	0.0444	4.8055	0.9992
M2	572	594.0522	622.6	0.022	5.2633	0.9996
M3	525	552.6044	558.0556	0.0428	4.3476	0.9993
M4	572	598.822	599.3529	0.0372	4.9085	0.9994
M5	540	574.9222	582.8448	0.0703	3.7531	0.9984
M6	562	579.2826	597.4545	0.0471	4.2469	0.9991
M7	539	564.3252	624	0.032	4.7163	0.9993
M8	582	598.3468	606.4737	0.0421	4.6507	0.9993
R1	292	361.2355	352.1538	0.0799	3.2486	0.9988
R2	267	329.0938	339	0.0735	3.2969	0.9989
R3	312	358.785	365.8235	0.0823	3.2541	0.999
R4	262	335.9728	333.5	0.0843	3.2057	0.9989
R5	273	324.8863	334	0.097	3.1313	0.999
R6	290	342.5008	364.2857	0.0819	3.2831	0.9989
R7	256	296.5531	290.1818	0.0835	3.246	0.9992
R8	279	347.5039	348.0303	0.0917	3.1508	0.9989

Table S3 KEGG function prediction

Class1	Class2	R: mean rel.freq.(%)	R: std.dev.(%)	M: mean rel.freq.(%)	M: std.dev.(%)	p-values	Difference between means
Metabolism	Carbohydrate metabolism	8.883439	0.04585	9.354409	0.232478	0.00133	-0.47097
Metabolism	Lipid metabolism	1.972809	0.005608	2.484798	0.071476	1.06E-06	-0.51199
Metabolism	Metabolism of cofactors and vitamins	4.638211	0.017373	3.984027	0.098868	1.11E-06	0.654183
Metabolism	Energy metabolism	4.383104	0.033685	4.144153	0.108688	0.000713	0.238951
Metabolism	Amino acid metabolism	6.434252	0.036154	8.000386	0.566679	0.000289	-1.56613
Metabolism	Nucleotide metabolism	3.924103	0.016772	2.866114	0.092703	3.35E-08	1.057989
Metabolism	Biosynthesis of other secondary metabolites	0.882654	0.004903	0.88302	0.027139	0.972938	-0.00037

Metabolism	Metabolism of terpenoids and polyketides	1.168176	0.005523	1.523482	0.139499	0.000469	-0.35531
Metabolism	Xenobiotics biodegradation and metabolism	0.954724	0.008444	2.20728	0.221507	4.60E-06	-1.25256
Metabolism	Metabolism of other amino acids	1.387329	0.002569	1.64227	0.049011	7.44E-06	-0.25494
Metabolism	Glycan biosynthesis and metabolism	1.534626	0.013536	0.97673	0.080903	1.04E-06	0.557896
Genetic Information Processing	Translation	3.894125	0.014996	2.615477	0.084175	5.79E-09	1.278648
Metabolism	Global and overview maps	43.32704	0.082044	42.22295	0.387132	0.000207	1.104093
Human Diseases	Drug resistance: Antimicrobial	0.924019	0.007067	0.783479	0.09786	0.009031	0.140539
Human Diseases	Drug resistance: Antineoplastic	0.023378	0.00125	0.071073	0.007861	2.10E-06	-0.04769

Environmental Information Processing	Membrane transport	3.709301	0.040312	3.900721	0.446984	0.316375	-0.19142
Environmental Information Processing	Signal transduction	1.792662	0.010323	2.4788	0.272473	0.000485	-0.68614
Cellular Processes	Cellular community - prokaryotes	1.392987	0.011539	1.684539	0.036605	1.27E-07	-0.29155
Cellular Processes	Cell motility	0.127005	0.008411	0.690011	0.135837	2.84E-05	-0.56301
Genetic Information Processing	Folding, sorting and degradation	1.475633	0.007072	1.235968	0.049861	9.55E-06	0.239665
Genetic Information Processing	Transcription	0.175784	0.000876	0.124811	0.002346	2.48E-11	0.050973
Genetic Information Processing	Replication and repair	3.15978	0.012569	2.186393	0.070303	9.54E-09	0.973387

Organismal Systems	Endocrine system	0.580516	0.00709	0.596796	0.01362	0.021525	-0.01628
Environmental Information Processing	Signaling molecules and interaction	0.044647	0.000258	0.028907	0.000695	2.75E-11	0.01574
Cellular Processes	Cell growth and death	0.529119	0.004051	0.497539	0.022647	0.009902	0.03158
Cellular Processes	Transport and catabolism	0.238787	0.001467	0.264106	0.010833	0.000658	-0.02532
Organismal Systems	Aging	0.369564	0.002708	0.370638	0.020652	0.91509	-0.00107
Organismal Systems	Circulatory system	0.042303	0.002542	0.027259	0.006609	0.000528	0.015044
Organismal Systems	Development	0	0	2.34E-06	2.76E-06	0.067347	########
Cellular Processes	Cellular community - eukaryotes	1.23E-05	7.06E-06	0.000139	9.17E-05	0.010408	-0.00013
Organismal Systems	Immune system	0.04596	0.002018	0.03424	0.004225	0.000127	0.011719
Organismal Systems	Environmental adaptation	0.109374	0.000585	0.115877	0.00775	0.067919	-0.0065
Organismal Systems	Nervous system	0.121119	0.002131	0.185374	0.037315	0.003621	-0.06425

Organismal Systems	Sensory system	4.77E-06	2.94E-06	5.10E-05	3.47E-05	0.012028	########
Human Diseases	Endocrine and metabolic diseases	0.215427	0.000996	0.177432	0.008002	1.04E-05	0.037994
Organismal Systems	Excretory system	0.013669	0.001053	0.026778	0.001432	7.14E-10	-0.01311
Organismal Systems	Digestive system	0.073867	0.001721	0.041369	0.008842	4.15E-05	0.032498
Human Diseases	Neurodegenerative diseases	0.281568	0.007427	0.29518	0.040799	0.430947	-0.01361
Human Diseases	Substance dependence	0.002846	0.000361	0.034114	0.010502	0.000199	-0.03127
Human Diseases	Infectious diseases: Bacterial	0.414652	0.002392	0.375867	0.035518	0.027521	0.038785
Human Diseases	Infectious diseases: Parasitic	0.041491	0.001687	0.075876	0.004792	1.83E-07	-0.03438
Human Diseases	Infectious diseases: Viral	0.003645	0.000464	0.045874	0.019346	0.001008	-0.04223
Human Diseases	Cancers: Overview	0.568158	0.002927	0.561623	0.006872	0.051201	0.006536
Human Diseases	Cancers: Specific types	0.062814	0.001721	0.130327	0.017221	3.67E-05	-0.06751
Human Diseases	Immune diseases	0.074552	0.001538	0.036889	0.001206	0	0.037663

Human Diseases	Cardiovascular diseases	0.000762	0.000134	0.016853	0.005799	0.0003	-0.01609

Table S4 COG function prediction

Class1	Class2	R: mean rel.freq.(%)	R: std.dev.(%)	M: mean rel.freq.(%)	M: std.dev.(%)	p-values	Difference between means
INFORMATION STORAGE AND PROCESSING	RNA processing and modification	0.026886	0.001262	0.040951	0.013555	0.032625	-0.01406
INFORMATION STORAGE AND PROCESSING	Chromatin structure and dynamics	0.033982	0.00098	0.039706	0.007425	0.084889	-0.00572
METABOLISM	Energy production and conversion	5.579638	0.037921	6.073492	0.216543	0.000763	-0.49385
CELLULAR PROCESSES AND SIGNALING	Cell cycle control, cell division, chromosome partitioning	1.459879	0.008335	1.009213	0.016381	1.67E-13	0.450666

METABOLISM	Amino acid transport and metabolism	7.834755	0.036495	9.030411	0.577256	0.001425	-1.19566
METABOLISM	Nucleotide transport and metabolism	3.475337	0.021065	2.52883	0.071375	2.17E-09	0.946507
METABOLISM	Carbohydrate transport and metabolism	5.835497	0.078328	6.762894	0.476146	0.001784	-0.9274
METABOLISM	Coenzyme transport and metabolism	4.977711	0.024751	4.416378	0.143993	2.94E-05	0.561333
METABOLISM	Lipid transport and metabolism	2.829175	0.015841	3.995049	0.637956	0.002618	-1.16587
INFORMATION STORAGE AND PROCESSING	Translation, ribosomal structure and biogenesis	8.728571	0.037099	5.658637	0.094822	2.46E-13	3.069934
INFORMATION STORAGE	Transcription	5.547268	0.062517	7.699707	0.194173	5.46E-09	-2.15244

AND PROCESSING							
INFORMATION STORAGE AND PROCESSING	Replication, recombination and repair	7.509548	0.034654	5.299041	0.310043	7.29E-07	2.210507
CELLULAR PROCESSES AND SIGNALING	Cell wall/membrane/envelope biogenesis	7.347763	0.035642	5.850639	0.113816	2.23E-09	1.497124
CELLULAR PROCESSES AND SIGNALING	Cell motility	0.851858	0.018862	1.367555	0.28939	0.002825	-0.5157
CELLULAR PROCESSES AND SIGNALING	Posttranslational modification, protein turnover, chaperones	4.04353	0.018692	3.565939	0.083344	1.73E-06	0.477592
METABOLISM	Inorganic ion transport and metabolism	5.528844	0.020279	5.049294	0.194984	0.00056	0.47955

METABOLISM	Secondary metabolites biosynthesis, transport and catabolism	1.188608	0.012821	2.640668	0.546117	0.000393	-1.45206
POORLY CHARACTERIZED	General function prediction only	11.15609	0.033805	12.01119	0.267418	0.000117	-0.8551
POORLY CHARACTERIZED	Function unknown	8.343026	0.032279	7.846619	0.289341	0.00309	0.496407
CELLULAR PROCESSES AND SIGNALING	Signal transduction mechanisms	3.107754	0.054443	5.251799	0.504357	2.15E-05	-2.14404
CELLULAR PROCESSES AND SIGNALING	Intracellular trafficking, secretion, and vesicular transport	2.521034	0.03548	2.037055	0.276141	0.002857	0.48398
CELLULAR PROCESSES	Defense mechanisms	2.045319	0.031111	1.811781	0.233852	0.036319	0.233538

AND SIGNALING							
CELLULAR PROCESSES AND SIGNALING	Extracellular structures	0.025516	0.001135	0.001373	0.001394	1.36E-13	0.024142
CELLULAR PROCESSES AND SIGNALING	Nuclear structure	0	0	0	0	1	0
CELLULAR PROCESSES AND SIGNALING	Cytoskeleton	0.002413	0.000233	0.011779	0.000903	1.76E-08	-0.00937
